# Comparative analysis of chemical and lentiviral approaches in the generation of human induced pluripotent stem cell–derived motor neurons

**DOI:** 10.4103/NRR.NRR-D-24-00435

**Published:** 2025-05-06

**Authors:** Masood Sepehrimanesh, Wu Xu, Baojin Ding

**Affiliations:** 1 Department of Biochemistry and Molecular Biology, Louisiana State University Health Sciences Center at Shreveport, Shreveport, LA, USA; 2 Department of Chemistry, University of Louisiana at Lafayette, Lafayette, LA, USA

**Keywords:** chemicals, human induced pluripotent stem cells, lentivirus, motor neuron diseases, motor neurons, movement disorders, neural progenitor cells, transcription factors

## Abstract

The generation of human induced pluripotent stem cell–derived motor neurons overcomes limited access to human tissues and offers an unprecedented approach to modeling motor neuron diseases such as dystonia and amyotrophic lateral sclerosis. Motor neurons generated through different strategies may exhibit substantial differences in purity, maturation, characterization, and even neuronal identity, leading to variable outcomes in disease modeling and drug screening. However, very few comparative studies have been conducted to determine the similarities and differences among motor neurons prepared via different protocols. In this study, we prepared human induced pluripotent stem cell–derived motor neurons via lentiviral delivery of transcription factors and chemical induction and performed a systematic comparative analysis. We found that motor neurons generated by both approaches showed typical motor neuron morphology and robustly expressed motor neuron-specific markers, such as nuclear homeobox transcription factor 9 and choline acetyltransferase. The chemical induction protocol utilizes a combination of small molecules to induce motor neuron differentiation, offering a significantly faster maturation time of 35 days compared to 46 days with lentiviral delivery of transcription factors. Additionally, while lentiviral delivery of transcription factors are suitable for downstream biochemical analysis, chemical induction are more applicable for therapeutic approaches as they avoid the use of lentiviruses. Both approaches produce motor neurons with high purity (> 95%) and yield. No significant differences were found between chemical induction and lentiviral delivery of transcription factors in terms of motor neuron markers and maturation markers. These robust methodologies offer researchers powerful tools for investigating motor neuron diseases and potential therapeutic strategies.

## Graphical abstract

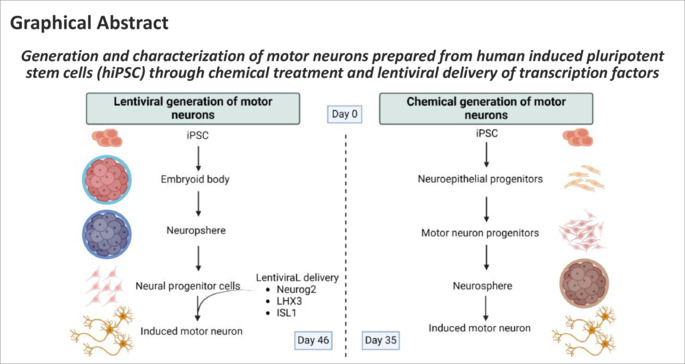

## Introduction

The generation of human motor neurons (MNs) holds significant importance in modeling movement disorders, drug screening, and developing cell therapy (Akter and Ding, 2022). MN diseases (MNDs), such as amyotrophic lateral sclerosis (ALS), are debilitating conditions characterized by the progressive degeneration of MNs. Because they can recapitulate cellular and molecular disease pathogenesis, hiPSC-MNs have emerged as powerful tools in modeling MNDs (Ding, 2021).

In both the human and mouse nervous systems, the process of MN development involves several consecutive stages, including MN generation, differentiation, and maturation. Neural stem cells divide during early stages to produce neural progenitor cells (NPCs), which have the potential to differentiate into various types of neurons, including excitatory and inhibitory neurons, as well as glial cells such as astrocytes and oligodendrocytes during embryonic and early postnatal stages *in vivo* (Llorente et al., 2022; Hosain and Ding, 2024). During embryonic development, MNs originate from NPCs located in the ventral region of the spinal cord, known as the ventral neural tube (Shaker et al., 2021). These progenitor cells are specified to become MNs through the action of various signaling molecules, including sonic hedgehog and expression of specific transcription factors, such as oligodendrocyte transcription factor 2 (OLIG2), NK6 homeobox 1 (NKX6.1), insulin gene enhancer protein (ISL1), and ISL2 (Sagner et al., 2018). After the generation of immature neurons, they undergo maturation processes to become fully functional. This includes the development of neuronal morphology, including dendritic arborization and axonal outgrowth. The synaptogenesis also occurs during this stage (Lewis et al., 2013).

Based on findings regarding the molecular mechanisms underlying MN differentiation and regulation *in vivo* (Ding, 2015), various techniques have been developed to generate patient-derived MNs *in vitro*. Patient-specific MNs can be generated either through direct conversion from skin fibroblast cells using lentiviral delivery of transcription factors (Sepehrimanesh et al., 2021) or through differentiation of hiPSC, which are reprogrammed from somatic cells by introducing Yamanaka factors (Takahashi et al., 2007). Advanced genome editing techniques enable the introduction of disease-causing mutations into healthy iPSCs and the correction of mutations in patient iPSCs (Akter et al., 2023), offering an unprecedented approach to creating disease models and isogenic controls for the investigation of disease mechanisms and therapeutic interventions. For instance, in a study by Alves et al. (2015), patient-derived hiPSC-MNs faithfully recapitulated key disease phenotypes of ALS, including aberrant protein aggregation and impaired axonal transport, offering valuable insights into ALS pathology. Additionally, Sances et al. (2016) demonstrated the utility of hiPSC-MNs in modeling spinal muscular atrophy, showing disease-specific phenotypes such as reduced survival and altered electrophysiological properties. These findings underscore the high fidelity of hiPSC-MNs in capturing disease-associated phenotypes, thus facilitating the progress of elucidation of disease mechanisms and the screening of potential therapeutic compounds.

Furthermore, hiPSC-MNs enable the study of patient-specific genetic mutations implicated in MNDs, offering a personalized approach to disease modeling. For example, Devlin et al. (2015) utilized hiPSCs derived from ALS patients carrying mutations in the *C9orf72* gene to generate MNs displaying characteristic pathological features, including RNA foci formation and nucleocytoplasmic transport defects. Similarly, Burkhardt et al. (2013) demonstrated the utility of hiPSC-MNs in modeling familial ALS caused by mutations in the superoxide dismutase type 1 (*SOD1*) gene, highlighting disease-specific phenotypes such as mitochondrial dysfunction and susceptibility to oxidative stress. Recently, we have generated human patient-specific MNs to model DYT1 dystonia, a movement neurological disease, and revealed disease-dependent cellular deficits, including disrupted neurodevelopment, deformed nucleus, impaired nucleocytoplasmic transport, and nuclear lamin-B1 dysregulation (Ding et al., 2021; Akter et al., 2024). All these studies underscore the versatility of hiPSC-MNs in capturing the genetic complexity of MNDs and provide a powerful platform for investigating disease mechanisms and developing targeted therapies.

In the field of drug screening, the generation of human MNs is instrumental in improving the efficiency and accuracy of drug development. Traditionally, drug discovery has relied heavily on animal models, which may not fully capture the complexity of human physiology due to species-dependent differences. However, the use of hiPSC-MNs offers a more relevant and predictive *in vitro* model. The utility of human MNs in drug screening for neurodegenerative diseases has been demonstrated in many studies (Takahashi et al., 2007; Choi et al., 2014; Okano et al., 2023; Beghini et al., 2024). This model allows researchers to evaluate drug efficacy, toxicity, and side effects specifically on human MNs, enabling the identification of potential therapeutics with higher precision and reducing the reliance on animal testing (Silva and Haggarty, 2020; Xiong et al., 2021). Also, this approach has the potential to accelerate the discovery and development of novel treatments for movement disorders and other neurological conditions. For instance, by using hiPSC-MNs, we discovered that RAN binding protein 17 (RANBP17) overexpression restored nuclear transport activity and ameliorated neurodevelopment of DYT1 dystonia MNs.

However, the research field of generating iPSC-MNs is facing several challenges. First, there is a lack of standardization in protocols, resulting in variations in reagents, treatments, and culture times across different studies. Various methods for generating hiPSC-MNs involve the use of small molecules, transcription factors, and combinations of inhibitors and growth factors. For instance, glycogen synthase kinase-3 beta and suppressor of mothers against decapentaplegic inhibitors, bone morphogenetic protein inhibitors, γ-secretase inhibitors, antioxidants, and insulin-like growth factor 1 are used to induce NPCs (Lin et al., 2017; Bianchi et al., 2018; Fujimori et al., 2018), while sonic hedgehog signaling activation and neurotrophic factors aid in MN maturation and survival (Ben-Shushan et al., 2015; Hall et al., 2017). Additionally, generating hiPSC-MNs using lentiviral delivery of transcription factors remains challenging due to difficulties in achieving efficient and reproducible viruses (Sepehrimanesh and Ding, 2020; Sepehrimanesh et al., 2021; Akter et al., 2022). This inconsistency hampers effective comparison and interpretation of results. Second, the quality of hiPSC-MNs generated by different methods varies significantly, complicating the characterization and interpretation of the data. It is crucial to optimize these processes to ensure the generation of iPSC-MNs faithfully represents their *in vivo* counterparts. Improving reliability and reproducibility is essential for robust research outcomes and potential clinical applications. Addressing these challenges is critical to enhancing the utility of hiPSC-MNs in both basic and translational research.

In this study, we systematically compared two widely used protocols for generating hiPSC-MNs, chemical induction (ciMNs) and lentiviral delivery of transcription factors (Lenti-MNs). Our focus was on evaluating the quality of MNs generated and their potential applications in basic and translational research. This study systematically compared the similarities and differences of MNs prepared by two approaches, providing valuable insights for neuroscience research utilizing iPSC-MNs as a model system.

## Methods

### Cell lines and culture conditions

The use of human cell lines, including human induced pluripotent stem cells (hiPSCs), in this study was approved by the Institutional Review Board (IRB) of Louisiana State University Health Sciences Center at Shreveport (LSUHSC-S, IRB ID: STUDY00002945). Human embryonic kidney 293T (HEK293T) cells were purchased from American Type Culture Collection (ATCC, Manassas, VA, USA, Cat# CRL-3216, RRID: CVCL_0063) and maintained in Dulbecco’s modified Eagle medium (DMEM; Gibco, Carlsbad, CA, USA) with 10% fetal bovine serum (FBS) and 1% Penicillin/Streptomycin (Pen/Strep) for future lentivirus preparation. hiPSC WT 1A2 was purchased from Coriell Institute for Medical Research (Camden, NJ, USA, Cat# GM23476, RRID: CVCL_T841). hiPSC maintained in mTeSR1 basal medium supplemented with mTeSR1 supplement (STEMCELL Technologies, Vancouver, Canada) and 1% Pen/Strep on Matrigel (Corning, Cat# 356234) coated plate at 37°C and 5% CO_2_ (Sepehrimanesh and Ding, 2020).

The animal work protocol in this study was approved by the Animal Care and Use Committee at LSUHSC-S (Proposal number: P-24-003). Mouse astrocytes were isolated from non-specific pathogen free (non-SPF) postnatal day 1 (P1) C57BL/6NCrl inbred pups, regardless of their gender, and cultured in DMEM with 15% FBS, and 1% Pen/Strep, as described previously (Schildge et al., 2013; Sepehrimanesh et al., 2021). Briefly, breeding was done between C57BL/6NCrl male and female (Charles River, Wilmington, MA, USA) in the ratio of 1:1. Pups were separated from parents after birth and under aseptic conditions and after decapitation, the brain was excised and washed with 70% ethanol followed by cold Hank’s Balanced Salt Solution (HBSS; Gibco) containing 10% Pen/Strep. The brain was then cut into small pieces, washed, and pipetted using different pipette sizes to create a homogeneous suspension. This cell suspension was seeded onto gelatin-coated 10 cm culture plates and incubated undisturbed for 3 days, allowing astrocytes to adhere to the bottom while other cells remained floating and eventually died. After 3 days, the medium was changed, and the cells were allowed to grow and expand until they reached full confluence.

### Plasmid and lentivirus preparation

Preparation and optimization of a third-generation lentiviral vector which co-expressed three transcription factors of neurogenin 2 (NEUROG2), Gene ID: 63973), ISL1 (Gene ID: 3670), and Lin11/Isl1/Mec3 (LIM) Homeobox 3 (LHX3, Gene ID: 8022) has been previously described (Sepehrimanesh and Ding, 2020). As previously described (Ding et al., 2013), HEK293T cells were utilized to generate replication-incompetent lentiviruses, and viral supernatants were harvested at 48 and 72 hours following transfection. The viral supernatants underwent filtration using 0.45 μm syringe filters. Subsequently, they were titrated according to established methods and stored at 4°C prior to cell transduction.

### The generation of human induced pluripotent stem cell–derived motor neurons using lentiviral delivery of transcription factors

This protocol was divided into two distinct phases: the generation of NPCs from hiPSCs and the conversion of NPCs to MNs. The generated NPCs can be preserved through freezing and subsequently utilized in the iMN generation step.

#### First step: Generation of neural progenitor cells from human induced pluripotent stem cells

NPCs were generated based on our previous report (Akter et al., 2022). Briefly, hiPSCs were cultured on Matrigel-coated 6-well plates in mTeSR1 medium containing mReSR1 basal media (STEMCELL Technologies), 1× of 5× supplement (STEMCELL Technologies), and Pen/Strep (Thermo Fisher Scientific, Waltham, MA, USA) until they reached 70% confluence. Neuroectodermal fate induction was achieved by adding 10 µM all-trans-retinoic acid (RA; MilliporeSigma, Burlington, MA, USA) and 0.5 mM valproic acid (VPA; Sigma) to the mTeSR1 medium for 7 days. The hiPSCs were then enzymatically dissociated by adding 1 mL Versene (Thermo Fisher Scientific) to each well and incubated at 37°C for 6–7 minutes. Versene and then carefully pipetted into small clumps. Additionally, 10 μM of Y-27632 Rock Inhibitor (STEMCELL Technologies) was added to inhibit apoptosis and increase cell survival. Cell clumps were aggregated in the resuspension growth/ KnockOut Serum Replacement (KOSR; Thermo Fisher Scientific) media containing Dulbecco’s modified Eagle’s medium/nutrient mixture F-12 (DMEM-F12, Hyclone, Washington, D.C., USA), 20% KOSR (Thermo Fisher Scientific), 1% glutamax (Thermo Fisher Scientific), 1% MEM Non-Essential Amino Acids Solution (NEAA, Thermo Fisher Scientific), 50 µM 2-mercaptoethanol (BME, Thermo Fisher Scientific) and 1× Pen/Strep for 4 days, followed by culturing in the neurosphere (NSP) medium containing DMEM/F12, 1× N2 supplement (Thermo Fisher Scientific), 1% Pen/Strep, 1% Glutamax, 1× NEAA, 50 μM BME, 8 μg/mL heparin (Thermo Fisher Scientific), 10 ng/mL heat stable recombinant human basic fibroblast growth factor (bFGF; Gibco), and 10 ng/mL human epidermal growth factor recombinant protein (EGF; Gibco) for another one week. Formed NSPs were subsequently dissociated into individual cells using 3 mL Accutase (Innovative Cell Technologies, San Diego, CA, USA). The dissociated NPCs were then cultured and expanded on Matrigel (Corning)-coated 6-well plates in NPC medium consisted of a 1:1 mixture of DMEM/F12 and Neurobasal medium (Thermo Fisher Scientific), supplemented with 0.5% N2 (Invitrogen, Waltham, MA, USA), 1% B27 (Invitrogen), 1% GlutaMax, 1% NEAA, 50 µM BME), 1% Pen/Strep, 10 ng/mL EGF, 10 ng/mL bFGF, and 1% Pen/Strep. Upon reaching approximately 90% confluence, the cells were preserved for long-term storage by freezing them in a freezing medium consisting of NPC medium supplemented with 10% dimethyl sulfoxide. The frozen cells were then stored in liquid nitrogen for future use.

#### Second step: Generation of human induced pluripotent stem cell–derived motor neurons

For lentiviral-assisted differentiation, NPCs were seeded onto plates coated with Matrigel at a density of 3 × 10^4^ cells/cm^2^ and allowed to adhere overnight (12–16 hours). Subsequently, the cells were infected with lentivirus that expressed three transcription factors with a multiplicity of infection (MOI) of approximately 2. On the following day, the medium was substituted with a neuronal maturation medium comprising 5 mM forskolin (FSK; MilliporeSigma), along with 10 ng/mL each of brain-derived neurotrophic factor (BDNF; PeproTech, Waltham, MA, USA), Glial cell line-derived neurotrophic factor (GDNF; PeproTech), and neurotrophin-3 (NT3; PeproTech). At 4 days post-infection (dpi), the neurons were dissociated using Accutase and subsequently replated onto coverslips seeded with astrocytes. The neurons were replated at a density of 1 × 10^3^ cells/cm^2^ to culturing for long term and having the mature MNs.

### Chemical induced motor neurons

ciMNs were generated following a modified version of the previously published report (Du et al., 2015). Briefly, after culturing hiPSCs on Matrigel-coated 6-well plates in mTeSR1 medium and achieving 70% confluence, the culture medium was subsequently replaced with six different media, each used separately. These media shared a common basic recipe (BR), which consisted of a 1:1 mixture of DMEM/F12 and Neurobasal medium supplemented with 0.5% N_2_, 0.5% B27, 0.1 mM L-ascorbic acid (L-AA; MilliporeSigma), 1% GlutaMAX, and 1% Pen/Strep. However, for each stage, different chemicals were added to the BR. Neuroepithelial progenitor cells (NEPs) were obtained by culturing hiPSCs in the BR medium supplemented with 3 μM CHIR99021 (Thermo Fisher Scientific), 2 μM DMH1 (Thermo Fisher Scientific), and 2 μM SB431542 (Thermo Fisher Scientific) for 6 days. The culture media were changed every other day during this period. Following the 6-day culture period, during which the morphology transformed into NEPs as confirmed by the expression of Vimentin and Sex determining region Y-box 2 (SOX2), the culture media were replaced with the BR medium supplemented with 1 μM CHIR99021, 2 μM DMH1 (Thermo Fisher Scientific), 2 μM SB431542 (Thermo Fisher Scientific), 0.1 μM RA, and 0.5 μM purmorphamine (PMA; Thermo Fisher Scientific). This medium was maintained for an additional 6 days, with media changes every other day, to generate early motor neuron progenitors (eMNPs) as confirmed by the expression of SOX2 and OLIG2 markers.

For an additional 3 days, the culture was maintained in the BR medium supplemented with 1 μM CHIR99021, 2 μM DMH1, 2 μM SB431542, 0.1 μM RA, 0.5 μM PMA, and 0.5 mM VPA. This medium was used to induce the differentiation of the eMNPs into mature motor neuron progenitors (mMNPs). mMNPs were subsequently enzymatically dissociated using Accutase and gently pipetted into small clumps. To prevent apoptosis and enhance cell survival, 10 μM Y-27632 Rock Inhibitor was added during the dissociation process. The small clumps of mMNPs were then cultured in suspension using MN differentiation media, composed of the BR supplemented with 0.5 μM RA and 0.1 μM PMA. The culture of the clumps in the MN differentiation media continued for an additional 6 days. The formed spheres were later dissociated into individual cells or small aggregates using Accutase. These dissociated MNs were then cultured and expanded on Matrigel-coated 6- or 24-well plates. The culture medium used for MN maturation consisted of the BR supplemented with 0.5 μM RA, 0.1 μM PMA, and growth factors including insulin-like growth factor 1 (Thermo Fisher Scientific), BDNF, and Ciliary neurotrophic factor (CNTF; Thermo Fisher Scientific) at a concentration of 10 ng/mL. This culture setup facilitated the generation of both early and mature MNs.

### Immunocytochemistry and imaging

Immunocytochemistry and capturing images were conducted following previously reported methods (Cui et al., 2022). The motor neurons were fixed at specified time points using 4% paraformaldehyde in phosphate-buffered saline (PBS) for 15 minutes at room temperature (RT). After fixation, they were permeabilized and blocked for 1 hour in a blocking buffer consisting of PBS with 0.2% Triton X-100 and 3% bovine serum albumin. Subsequently, the cells were incubated overnight at 4°C with primary antibodies diluted in the blocking buffer. The primary antibodies used in this study were:

• Mouse anti-choline acetyltransferase (ChAT) (1:200, DSHB, Framingham, MA, USA, Cat# ChAT4B1-s): A mature MN marker catalyzing the biosynthesis of acetylcholine, regulating signal transduction at the neuromuscular junction (Ding et al., 2020).

• Mouse anti-homeodomain protein (HB9) (1:500, DSHB, Cat# 81.5C10, RRID: AB_2145209): Also known as motor neuron and pancreas homeobox 1 (MNX1), a transcription factor described as a spinal cord MN-specific marker and critical factor for the postmitotic specification of MNs (Letchuman et al., 2022).

• Mouse anti-tubulin beta-3 (TUBB3; 1:3000, BioLegend, San Diego, CA, USA, Cat# 801201, RRID: AB_2313773): A classic neuron marker involved in peripheral axon regeneration (Latremoliere et al., 2018).

• Chicken anti-microtubule-associated protein 2 (MAP2; 1:10,000, Abcam, Cat# ab5392, RRID: AB_2138153): A mature neuronal marker (Bai et al., 2023).

• Mouse anti-synaptotagmin-1 (SYT1) (1:250, DSHB, Cat# znp-1, RRID: AB_528483): A calcium-sensing protein resident in synaptic vesicles and an indicator of synapse formation (Lawrence et al., 2024).

• Chicken anti-Vimentin (1:2000, Cat# ab24525, Abcam). • Rabbit anti-Oligo2 (1:1000, Cat# NBP128667SS, Thermo Fisher Scientific).

• Mouse anti-SOX2 (1:200, Cat# sc-365823, Santa Cruz Biotechnology, Dallas, TX, USA).

Following primary antibody incubation, the cells were washed and incubated with corresponding secondary antibodies conjugated with fluorophores (1:250) for 2 hours at room temperature. All secondary antibodies (1:250) were purchased from Jackson ImmunoResearch (West Grove, PA, USA): donkey anti-mouse IgG AF488 (Cat# 715-545-150), sheep anti-mouse IgG Dylight 594 (Cat# 515-515-062), donkey anti-chicken IgY (IgG) AF488 (Cat# 703-545-155), and donkey anti-rabbit AF 594 (Cat# 711-585-152). 4′,6-Diamidino-2-phenylindole (DAPI; Thermo Fisher Scientific) was used to counterstain the cell nuclei. Confocal images were acquired using a Leica TCS SP5 Confocal microscope (Lica Company, Deerfield, IL, USA) equipped with a 63X/1.4 NA objective. Neurite length was quantified using ImageJ software version 1.54d (National Institutes of Health, Bethesda, MD, USA) and represented as the relative length per neurite, following the methodology described previously (Ding et al., 2013; Akter et al., 2024). Primary branches per cell were counted for neurites directly connected to the soma. Signals from DAPI staining were utilized to distinguish between the nucleus and cytoplasm.

### Quantitative reverse transcription-polymerase chain reaction

As previously described (Ding et al., 2021), total RNA was extracted from ciMNs at day 32 and Lenti-MNs at 21 dpi using phenol/chloroform extracting method. cDNA was synthesized using 0.5 µg of RNA from each sample with the SuperScript III First-Strand kit (Life Technologies, Carlsbad, CA, USA) and random hexamer primers. Quantitative reverse transcription-polymerase chain reaction (qRT-PCR) was conducted in triplicate using SYBR Green SuperMix (Invitrogen) and the BIO-RAD CFX-96 Fast Real-Time PCR system (Bio-Rad, Hercules, CA, USA), following standard protocols. Target mRNA levels were normalized to the reference gene GAPDH using the 2^–ΔCt^ method, which compares the cycle threshold (Ct) values of the target gene with those of the reference gene. The sequences of qRT-PCR primers can be found in **Table 1**.

**Table 1:** Evaluated genes and related sequences of primers

Gene	Primer sequence	Product size (bp)
*HB9*	F: 5′-GCA CCA GTT CAA GCT CAA C-3′	128
	R: 5′-GCT GCG TTT CCA TTT CAT CC-3′	
*ChAT*	F: 5′-ACA ACC ACG GAG ATG TTC TG-3′	115
	R: 5′-TGC AGC TGT GAA AGC TAG AG-3′	
*RBFOX3*	F: 5′-CTT ACG GAG CGG TCG TGT AT-3′	184
	R: 5′-TCA CAT GGT TCC AAT GCT GT-3′	
*SLC17A7*	F: 5′-AAA CAT GCT GAT CCC CTC AG-3′	170
	R: 5′-AAC CAC AAA AGG CTG TCG TC-3′	
*SLC17A6*	F: 5′- AGG GAC TTG TTG AGG GTG TG-3′	154
	R: 5′- CTG CAC AAG AAT GCC AGC TA-3′	
*GAPDH*	F: 5′-CAA ATT CCA TGG CAC-3′	133
	R: 5′-GGA CTC CAC GAC GTA CTC AG-3′	

ChAT: Choline acetyltransferase; F: forward; GAPDH: glyceraldehyde 3-phosphate dehydrogenase; HB9: homeobox gene 9; R: reverse; RBFOX3 (NeuN): RNA binding fox-1 homolog 3; SLC17A6 (VGLUT2): solute carrier family 17 member 6; SLC17A7 (VGLUT1): solute carrier family 17 member 7.

### Statistical analysis

Statistical analysis was performed using GraphPad Prism 8 (GraphPad Software, San Diego, CA, USA, www.graphpad.com). The D’Agostino & Pearson omnibus normality test was conducted first to determine if the data are normally distributed. If the data passed the normality test, independent samples *t*-test (for two groups), or one-way or two-way analysis of variance (three or more independent groups) was used to determine significance. If the data did not pass the normality test, the Kruskal-Wallis test was used to determine significance. Results are expressed as mean ± SEM of at least three biological replicates, and *P* < 0.05 is considered statistically significant.

## Results

### Generation of chemical induced motor neurons from human induced pluripotent stem cells

**Figure 1:**
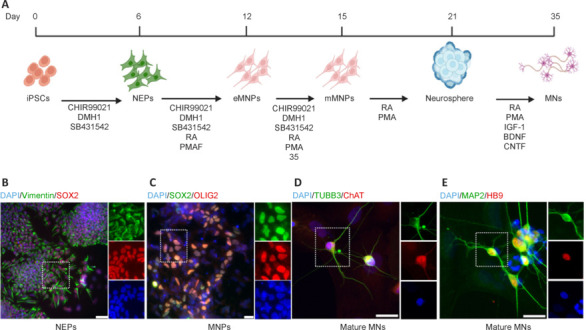
Generation of chemical induced MNs from hiPSC (ciMNs). (A) A schematic shows the process of the generation of ciMNs. The age of ciMNs was counted from the initiation of induction iPSC. (B) A representative micrograph of NEPs. DAPI stained nuclei. Scale bar: 20 µm. (C) A representative micrograph of MNPs. DAPI stained nuclei. Scale bar: 20 µm. (D) A representative micrograph of ciMNs on day 35. DAPI stained nuclei. Scale bar: 20 μm. (E) A representative micrograph of ciMNs on day 35. Scale bar: 20 µm. All experiments run at least three times. Schematic images were adapted from BioRender.com (2023). BDNF: Brain-derived neurotrophic factor; ChAT: choline acetyltransferase; ciMNs: chemical induced motor neurons from human induced pluripotent stem cells; CNTF: ciliary neurotrophic factor; DMH1: dorsomorphin homolog 1; eMNPs: early-stage motor neuron progenitors; HB9: homeodomain protein; IGF-1: insulin-like growth factor 1; MAP2: microtubule-associated protein 2; mMNPs: matured motor neuron progenitors; NEPs: neuroepithelial progenitors; NPCs: neuronal progenitor cells; OLIG2: oligodendrocyte transcription factor 2; PMA: purmorphamine; RA: retinoic acid; TUBB3: tubulin beta 3; VPA: valproic acid.

The process of generating ciMNs replicates the developmental stages during MN differentiation. It spans approximately 5 weeks and comprises several distinct stages, including the expansion of hiPSCs, the formation of NEPs, the transition to eMNPs, the maturation into mMNPs, the formation of neuron spheres, and ultimately the differentiation into mature MNs (**Figure 1A**). At each stage, cell identity can be confirmed by the expression of specific markers. For instance, cytoplasmic vimentin and nuclear SOX2 are highly expressed in NEPs (**Figure 1B**), and the co-expression of nuclear SOX2 and OLIG2 defines the MNP identity (**Figure 1C**). In addition to the generic neuron markers of TUBB3 and MAP2, the identity of MNs can be validated by the nuclear expression of MNX1 (HB9) in early development stages and ChAT in late maturation (**Figure 1D** and **E**). These neurons exhibited typical MN morphology with long axons. Based on the typical MN morphology and the expression of MN-specific markers HB9 and ChAT, we concluded that we have successfully generated hiPSC-MNs using chemical induction methods.

### Characterization of chemical induced motor neurons from human induced pluripotent stem cells

**Figure 2:**
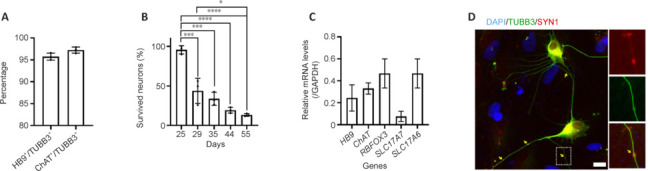
Characterization of ciMNs. (A) The purity of ciMNs shown as percentages of HB9^+^/TUBB3^+^ at 29 days, and ChAT^+^/TUBB3^+^ at 35 days. *n* (neurons) = 224 and 176 from triplicates, respectively. (B) Survival assay of ciMNs. The number of neurons on day 4 post-digestion (day 25 from initiation) was set as 100%. *P < 0.05, ***P < 0.001, ****P < 0.0001. One-way analysis of variance with Tukey’s *post hoc* test was used for statistical analysis. (C) Quantitative reverse transcription-polymerase chain reaction assay shows the relative mRNA levels of indicated markers in ciMNs on day 32. (D) Expression of the presynaptic marker synaptotagmin 1 (SYT1) at 35 days. The yellow arrows indicate SYT1^+^ puncta. Scale bar: 20 µm. All experiments run at least three times. ChAT: Choline acetyltransferase; ciMNs: chemical induced motor neurons from human induced pluripotent stem cells; HB9: homeobox gene 9; RBFOX3 (NeuN): RNA binding fox-1 homolog 3; SLC17A6 (VGLUT2): solute carrier family 17 member 6; SLC17A7 (VGLUT1): solute carrier family 17 member 7.

To assess the purity of ciMNs, we quantified the ratios of HB9-positive cells or CHAT-positive to TUBB3-positive cells (all neurons). Both ratios (HB9^+^/TUBB3^+^ and ChAT^+^/TUBB3^+^) exceed 95% (**Figure 2A**), indicating almost all neurons exhibit MN identity. Achieving survival poses a significant challenge when culturing induced MNs, particularly in achieving late maturation stages. To further characterize the survival of ciMNs, we conducted a survival assay, following an established protocol (Ding et al., 2021). CiMNs were replated onto coverslips with a monolayer of astrocytes, and the number of neurons on day 25 (4 days after replating) was set as 100%. The survival of neurons was calculated at various time points based on TUBB3 staining. Although the newly generated ciMNs were co-cultured with astrocytes, which are known to significantly support neuronal survival, the number of surviving neurons decreased noticeably over time. Approximately 50% of neurons died within the first few days after replating (day 8 *vs*. day 4 after replating) (**Figure 2B**). On day 55, only about 15% of the originally seeded neurons remained viable (**Figure 2B**), suggesting the significant challenge of maintaining long-term neuron cultures.

To further characterize the maturation of ciMNs, RNA was collected from pure cultured ciMNs at 32 days and subjected to quantitative PCR. Constantly, ciMNs robustly expressed the neuronal marker RNA binding fox-1 homolog 3 (RBFOX3) and MN markers of HB9 and CHAT (**Figure 2C**). Interestingly, at this stage, ciMNs exhibited significant expression of genes encoding subunits of vesicular glutamate transporters solute carrier family 17 member 7 (SLC17A7) and SLC17A6 (**Figure 2C**), indicating their progression towards functional maturation. On day 35, ciMNs highly expressed the presynaptic marker SYT1 and exhibited the synaptic puncta within neuronal processes (**Figure 2D**), suggesting that ciMNs had achieved functional maturation. These results indicate that ciMNs exhibited typical MN morphology, expressed specific MN markers, and gradually achieved functional maturation around day 35, although the survival rate and total yield were moderate.

### Generation and characterization of human induced pluripotent stem cell–derived motor neurons through lentiviral delivery of transcription factors

**Figure 3:**
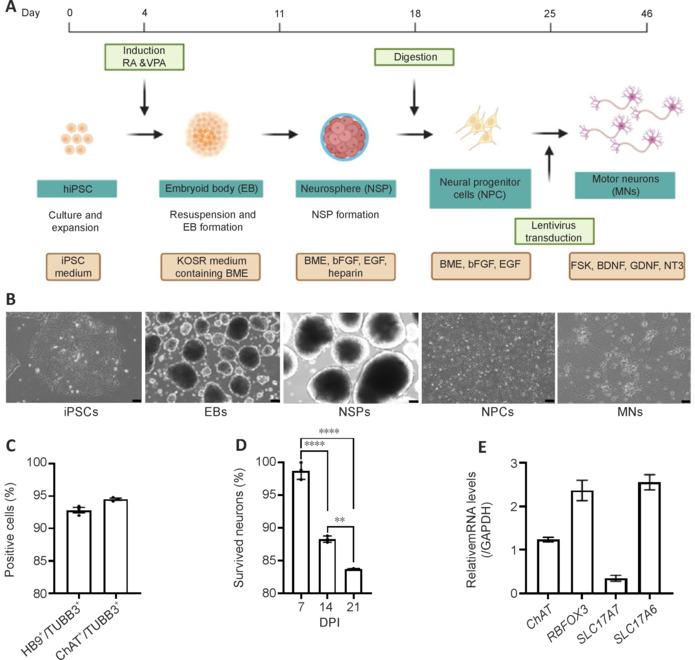
Generation and characterization of Lenti-MNs through lentiviral delivery transcription factors. (A) A schematic shows the process of the generation of human motor neurons (MNs) from hiPSCs. The age of Lenti-MNs was counted from the induction of iPSCs. (B) Representative micrographs of cells at each stage. Scale bars: 20 µm. (C) The purity of Lenti-MNs shown as percentages of HB9^+^/TUBB3^+^ and ChAT^+^/TUBB3^+^ neurons. Number of neurons (*n*) > 200 from triplicates. (D) Survival assay of lentivirus-induced hiPSC-MNs. The number of neurons at 7 dpi was set as 100%. **P < 0.01, ****P < 0.0001. One-way analysis of variance with Tukey’s *post hoc* test as a *post hoc* test was used for statistical analysis. (E) Quantitative reverse transcription-polymerase chain reaction assay shows the relative gene expression levels of different markers in Lenti-MNs at 21 dpi. All experiments run at least three times. BDNF: Brain-derived neurotrophic factor; bFGF: basic fibroblast growth factor; BME: beta mercaptoethanol; ChAT: choline acetyltransferase; EB: embryoid body; EGF: epidermal growth factor; FSK: forskolin; GDNF: Glial cell line-derived neurotrophic factor; KOSR: knockout serum; NPC: neuronal progenitor cell; NSP: neurosphere; NT3: neurotrophic factor 3; RA: retinoic acid; RBFOX3 (NeuN): RNA binding fox-1 homolog 3; SLC17A6 (VGLUT2): solute carrier family 17 member 6; SLC17A7 (VGLUT1): solute carrier family 17 member 7; VPA: valproic acid.

In contrast, the lentiviral method for generating hiPSC-MNs (Lenti-MNs) showed a different trajectory. Initially, hiPSCs are induced to form embryoid bodies (EBs), which then differentiate into NSPs, and subsequently into NPCs (**Figure 3A**). At each stage, the cells show distinct morphological characteristics under different defined culture conditions (**Figure 3B**) (Sepehrimanesh and Ding, 2020; Akter et al., 2022; Akter and Ding, 2022). Subsequently (on around day 25), NPCs will be transduced with lentivirus expressing three transcription factors ISL-1, Neurog-2, and LHX3. These three factors are necessary and sufficient to induce the differentiation of NPCs into MNs (Sepehrimanesh and Ding, 2020). As early as 3 days post-viral infection (dpi), the infected NPCs exhibit an MN-like morphology with pronounced exons (**Figure 3B**). These Lenti-MNs robustly express MN markers HB9 and ChAT within 14 dpi and reach full maturation around 21 dpi (around 46 days from hiPSC culture). The purity of Lenti-MNs is over 90%, based on the ratios of HB9^+^/TUBB3^+^ and ChAT^+^/TUBB3^+^ (**Figure 3C**). We quantified the surviving neurons at 1, 2, and 3 weeks post-viral infection (wpi), with the numbers of neurons at 1 wpi set at 100%. We found that approximately 50% of Lenti-MNs survived at 2 wpi and about 20% survived at 3 wpi (**Figure 3D**). In addition to MN markers (ChAT), Lenti-MNs also robustly express maturation markers (RBFOX3, SLC17A7, and SLC17A6) at 21 dpi (**Figure 3E**), validating the MN identity and maturation.

### Comparison of chemical induced motor neurons from human induced pluripotent stem cells and Lenti-motor neurons

**Figure 4:**
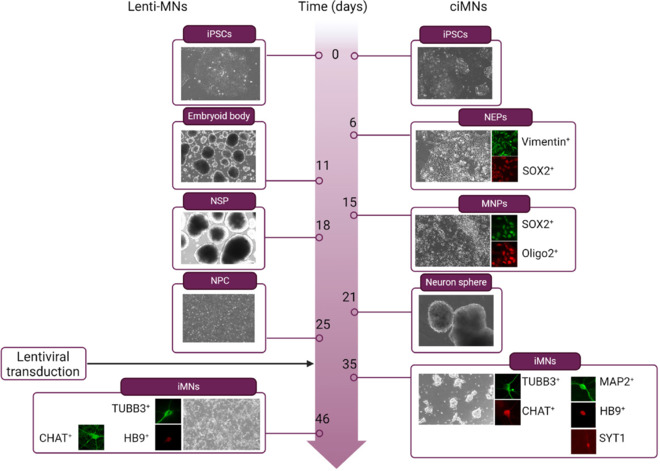
Comparison of two protocols in the generation of human induced pluripotent stem cell (hiPSC)–derived motor neurons (MNs). Left, The process of Lenti-MNs generation. Right, The process of ciMNs generation. In each process, representative images of cells at indicated time points were shown. These include iPSC, embryoid body, neurosphere (NSP), neural progenitor cells (NPCs), and induced motor neurons (iMNs) for Lenti-MNs generation. While for ciMNs generation, the steps include iPSC, neuroepithelial progenitors (NEP), motor neuron progenitors (MNPs), neuron sphere, and finally iMNs. Schematic images were created with BioRender.com.

The comparative analysis of the timelines for the two protocols revealed that both methods took more than one month from hiPSCs to mature MNs. Compared to Lenti-MNs, ciMNs achieved maturation relatively faster (35 days *vs*. 46 days) (**Figure 4**). However, the lentivirus-based method possesses a unique advantage that cells at the NPC stage can be prepared as frozen stocks. Depending on the desired experiments, these NPC frozen stocks can be thawed and expanded, rendering this protocol more feasible and manageable. In our attempts with the ciMNs method, we tried freezing and subsequently culturing them again from various intermediate stages, but the results did not meet our expectations.

The main similarities and differences between these two methods are summarized in **Table 2**. While Lenti-MNs utilize lentiviral delivery of transcription factors as induction agents, ciMNs rely on a cocktail of chemical compounds such as CHIR, SB, PMA, RA, and VPA to induce MN differentiation. Additionally, Lenti-MNs involve a series of steps including resuspension, digestion, and lentiviral transduction, while ciMNs require only resuspension and dissociation treatments, simplifying the process. Moreover, Lenti-MNs entail a digestion step on NSPs to generate NPCs, whereas ciMNs involve a dissociation step on MNPs to generate MNs. This variance contributes to differences in the time required for maturation, with Lenti-MNs taking **~**46 days from hiPSCs and **~**21 days from lentivirus transduction, compared with ciMNs’ **~**35 days from hiPSCs and **~**20 days from MNPs. Despite these differences, both methods share common goals and applications, including disease modeling, drug discovery, and potential use in cell therapy. However, each method comes with its own set of advantages and disadvantages. For instance, Lenti-MNs offer reproducibility through freezing stocks at the NPC stage, while ciMNs avoid the use of lentivirus. These advantages must be balanced against challenges such as the need for high-quality lentivirus in Lenti-MNs and variations due to chemical instability in ciMNs.

**Table 2:** Comparisons of chemical and lentiviral protocols for the generation of induced motor neurons (MNs)

	Lenti-MNs	ciMNs
Induction factors	Lentiviral delivery of transcription factors	Chemicals e.g., CHIR, SB, PMA, RA, VPA
Special treatment	Resuspension, Digestion, and Lentiviral transduction	Resuspension and dissociation
Digestion/dissociation	From NSPs to NPCs	From MNP to MNs
Time to maturation	46 days from hiPSC, and 21 days from lentivirus transduction	35 days from hiPSC, and 29 days from MNPs
Yield*	Excellent	Good
Purity**	90%	95%
Potential applications	Disease modeling, drug discovery, biochemical analysis	Disease modeling, drug discovery, cell therapy
Critical factors	Quality of NPCs and lentivirus	Concentration and stability of chemical compounds
Advantages	Freezing stocks at NPC stage make the protocol more feasible and reproducible	Relative homogenous treatment and avoid using lentivirus
Disadvantages	Need high-quality lentivirus	Variations due to the instability of chemicals

* MN yield is determined by the number of MNs that could be obtained from one million NPCs (for Lenti-MNs) or MNPs (for ciMNs). ** MN purity is defined by the percentage of HB9+ neurons among TUBB3+ cells (HB9+/TUBB3+) and the percentage of ChAT+ neurons among TUBB3+ cells (ChAT+/TUBB3+). ChAT: Choline acetyl transferase; ciMNs: chemical induced motor neurons from human induced pluripotent stem cells; ISL-1: insulin gene enhancer protein 1; LHX3: Lin11/Isl1/Mec3 (LIM) Homeobox 3; MNP: motor neuron progenitor; Neurog-2: neurogenin-2; NPC: neural progenitor cell; NSP: neurosphere; PMA: purmorphamine; RA: retinoic acid; TUBB3: tubulin beta-3; VPA: valproic acid.

## Discussion

The generation of hiPSC-MNs overcomes limited access to human brain tissues and offers an unprecedented approach to modeling MNDs. MNs generated through different strategies may exhibit substantial differences in purity, maturation, characterization, and even neuronal identity, resulting in variable outcomes in disease modeling and drug screening. However, very few comparative studies have been conducted to determine the differences and similarities among MNs prepared via different protocols. This study systematically compared the similarities and differences of MNs prepared by two approaches, thereby providing valuable insights for neuroscience research utilizing iPSC-MNs as a model system.

Ensuring both high purity and yield of hiPSC-MNs is crucial for advancing stem cell and neuroscience research. Scientists emphasize the importance of exploring diverse methodologies to optimize the generation of hiPSC-derived cell populations (Hester et al., 2011; Thiry et al., 2022; Limone et al., 2023). Our study provided a thorough examination and comparison of two different methods, ciMNs and Lenti-MNs, in the generation of hiPSC-MNs. By investigating these distinct approaches, this research sheds light on their respective efficiencies and potential advantages or limitations. We observed that the generation of ciMNs is significantly quicker compared to Lenti-MNs, taking approximately five weeks from the hiPSCs stage to obtain MNs, whereas Lenti-MNs require at least ten additional days. This time difference between the two procedures may be related to the equal distribution of chemical compounds in the culture media and better access of cells to required growth factors in ciMNs compared to Lenti-MNs. An obvious divergence in the timeline of different iPSC-induction protocols is reported from 30 days to nearly 7 weeks depending on the factors used (Faravelli et al., 2014). Park et al. (2016) have further expedited this procedure, successfully generating MNs from ALS-related mouse iPSCs in a remarkable timeframe of just 26 days.

We also verified cell identity at each intermediate stage using specific markers such as cytoplasmic vimentin and nuclear SOX2 for NEPs, and nuclear SOX2 and OLIG2 for MNPs. Vimentin emerges early as an intermediate filament in embryonic NEPs (Donato and Vickaryous, 2022). NEPs hold the potential for concurrent generation of both neuronal and glial cell lineages. *In vivo*, NEPs may differentiate to a vimentin-expressing stage, potentially implicating vimentin in cellular movements and interkinetic nuclear migration (Chen et al., 2023). Additionally, SOX2 stands out as one of the initial quartet of Yamanaka pluripotency factors, contributing to the preservation of neural progenitor populations (Donato and Vickaryous, 2022). NEPs also are pluripotent and express SOX2 as the pluripotency marker which acts as a molecular switch to regulate properties of neural stem cells (Takanaga et al., 2009; Shimozaki, 2014). Furthermore, MNPs represent another progenitor stage in this procedure and constitutively express SOX2 to maintain their neural fate and suppress their ability to differentiate into oligodendrocytes (Hoffmann et al., 2014; Zhang et al., 2018). While MNPs initially exhibit high levels of OLIG2 expression, their levels diminish over time. OLIG2 serves to sustain the MNP state, preserving them as a reservoir for oligodendrocyte differentiation, contrasting with Neurog2, which drives the conversion of MNPs into post-mitotic MNs (Lee et al., 2005).

Quantitative PCR analysis at day 32 confirmed the expression of MN markers HB9 and ChAT in ciMNs, along with RBFOX, SLC17A7, and SLC17A6. HB9, also known as MNX1, is selectively expressed by MNs (Lim et al., 2006) and plays a vital role in solidifying the fate of spinal MNs during development (Koronfel et al., 2021). While ChAT is present in all cholinergic neurons, including lower MNs, its expression is subdued in young lower MNs, making it an unreliable marker for young MNs but a dependable indicator for mature ones (Sances et al., 2016). The high expression level of RBFOX, SLC17A7, and SLC17A6 also indicate the maturation of ciMNs in our study. RBFOX3, also known as neuronal nuclei (NeuN), is a hallmark of post-mitotic neurons and promotes neuronal differentiation, playing essential roles in brain development and function (Wang et al., 2015). Its controlled splicing scheme is significant for the structural and functional development of postmitotic MNs (Jacko et al., 2018). SLC17A7 and SLC17A6 serve as markers for glutamatergic neurons and glutamatergic synapses (Du et al., 2020), facilitating the reuptake of glutamate into synaptic vesicles at excitatory presynaptic nerve terminals (Hu et al., 2020; Serrano-Saiz et al., 2020). Furthermore, ciMNs expressed SYT1 as a mature neuron marker. SYTs are a family of integral membrane proteins located on synaptic vesicles crucial for vesicular trafficking. SYT1 binds calcium ions, facilitating the initiation of neurotransmitter release at the synapse and is recognized as a key regulator enabling neurotransmitter release in the human brain (Ullah et al., 2021). SYT1 expression in ciMNs suggests the development of functional synaptic connections within the ciMN populations.

Despite focusing on timelines and specific markers, ensuring the high purity of generated MNs is paramount. To compare the purity of MNs generated using both methods, we stained iMNs for TUBB3, HB9, and ChAT. Our data indicated that the majority of neurons in both methods exhibited MN characteristics, suggesting the effectiveness of both protocols. For a more detailed description of the mechanisms underlying ciMNs, we utilized CHIR as a WNT agonist to promote neural induction, neuroepithelial proliferation, and MNP specification (Du et al., 2015). Additionally, we employed RA as a neurogenic compound and PMA as a sonic hedgehog-signaling agonist to aid in the generation of mixed ventral progenitors (Du et al., 2015). RA is known to initiate differentiation into various neuronal subtypes, while PMA enhances the differentiation of RA-treated cells specifically into MNs (Lin et al., 2020). Furthermore, we added two inhibitors of dorsalizing bone morphogenetic protein signaling, SB431542, and DMH1, to further direct the MN fate of the cells (Du et al., 2015). In the case of Lenti-MNs, we delivered three transcription factors, Neurog2, ISL1, and LHX3, using a third-generation lentivirus. Neurog2, a neural-specific transcription factor, plays a crucial role in specifying a neural fate and is expressed in NPCs within the developing central and peripheral nervous systems. ISL1, a member of the LIM/homodomain family of transcription factors, is required for MN generation, while LHX3 is a transcription factor necessary for MN specification (Sepehrimanesh and Ding, 2020).

We observed a decrease in survival over time in both methods. Despite utilizing different extracellular matrices to support neuron attachment, supplementing the culture medium with neurotrophic factors, and facilitating co-culture with glial cells like astrocytes, promoting survival in neuronal cultures (Sepehrimanesh and Ding, 2020), this decline in neuronal survival is inevitable. Furthermore, neurotrophic substances and growth factors, including BDNF, GDNF, and NT3 for Lenti-MNs, and BDNF and CNTF for ciMNs, were additionally employed to promote the growth, development, and viability of MNs (Akter et al., 2022). However, despite these efforts, the decrease in survival over time persists. This may be attributed to several factors such as the potential toxic effects of lentivirus and the challenge of achieving the superior support provided by various cell types *in vivo*.

A comparison between ciMNs and Lenti-MNs for MN generation reveals distinct differences in various aspects. ciMNs are induced using factors such as CHIR, SB, PMA, RA, and VPA, while Lenti-MNs are generated through lentiviral transduction of ISL-1, Neurog-2, and LHX3 transcription factors. Both methods involve six different culture media and special treatments of resuspension and digestion. However, ciMNs reach maturation in 35 days compared to 46 days for Lenti-MNs. In terms of yield, Lenti-MNs exhibit a high yield, while ciMNs show a moderate yield. However, ciMNs demonstrate slightly higher purity compared to Lenti-MNs. The potential applications of ciMNs lie in therapeutic approaches, whereas Lenti-MNs are more suitable for downstream biochemical analysis. Limitations of the protocols include the costliness of the chemical induction method and the inability to apply Lenti-MNs in treatment settings or for biochemical studies.

We acknowledge that there are some limitations in this study. First, we have only examined generic characterizations of iPSC-MNs, such as morphology, the expression of specific markers, and mature genes. However, neuronal functions such as electrophysiology, neurotransmitter release, and response to stimuli have not been fully characterized. These functional assays will provide novel insights into the differences between Lenti-MNs and ciMNs and offer guidelines for choosing the proper approach for generating iPSC-MNs in research. Second, we do not yet understand the possible molecular mechanisms underlying the differences between Lenti-MNs and ciMNs. Conducting transcriptomic studies would be valuable in revealing the differences in gene expression of Lenti-MNs and ciMNs at different developmental stages. Third, we did not compare the pathological differences between Lenti-MNs and ciMNs in modeling a specific movement disorder. MNs generated through different approaches may exhibit varying disease-dependent pathogenic manifestations, including differences in disease onset, cellular dysfunction, and molecular dysregulation. Further investigation into these differences would provide highly valuable insights for selecting the appropriate approach for disease modeling and drug screening using hiPSC-MNs.

In conclusion, we have provided a comprehensive comparison of ciMNs and Lenti-MNs, shedding light on both their shared characteristics and distinctive features. The observed similarities in marker expression and synaptic marker development underscore the robustness of both methods in generating functional MNs. The identified differences, including survival rates, maturation timelines, yield, purity, and practical considerations, offer nuanced insights for researchers in selecting an appropriate protocol based on their specific experimental goals and resource constraints. This work contributes valuable information to the scientific community, aiding in the optimization and informed selection of hiPSC-MN generation methods for diverse research applications.

## Data Availability

*No additional data are available*.
